# Candida auris in the Healthcare Environment: Prevalence, Anti-Fungal Resistance, and Survival on Porous & Non-Porous Surfaces

**DOI:** 10.1017/ash.2024.229

**Published:** 2024-09-16

**Authors:** Brandon Smith, Richard Miller

**Affiliations:** Environmental Safety Technologies, Inc.

## Abstract

Candida auris is an emerging multidrug-resistant pathogenic yeast capable of causing severe illness in the healthcare environment. It spreads easily amongst patient populations, is often resistant to anti-fungal treatments and can survive on surfaces for prolonged periods. In the current study, 85 sites within hospital settings were screened for surface-contaminated Candida species and C. auris. Surface swab samples were transferred to chromogenic agar media designed to isolate and identify Candida species and were incubated at 35°C for 48 hr. Samples were confirmed using molecular techniques designed to specifically target C. auris from other Candida species. Data was compiled to show prevalence of six key Candida species (C. albicans, C. auris, C. glabrata, C. krusei and C. tropicalis). Survivability on surfaces was performed using CDC B11903 C. auris strain. Plastic, metal and fabric surfaces used were purchased from a medical supply store. Once inoculated with 500 CFU/ml in sterile distilled water, the surfaces were kept in a Class II hood with minimal airflow and ambient conditions (21°C, 60% RH) and sampled daily. Results showed 25 of the 85 (29.4%) tested sites were positive for Candida species, with 3 of those sites positive for C. auris. Anti-fungal resistance among the three isolates (tested using concentration gradient test strips) showed notable resistance to fluconazole, but not to amphotericin B nor micafungin. C. auris survivability was dependent upon surface type, with the C. auris test strain surviving for 39 days on three different types of hospital curtains, and ≥10 days on a variety of non-porous plastic or metal surfaces. With demonstrated survivability of C. auris for long periods of time on hospital surfaces, it becomes critical for healthcare facilities to consider C. auris when developing infection prevention programs.

